# CD14 as A Serum Immune Biomarker and Genetic Predisposition Factor for Allergic Rhinitis

**Published:** 2019-01

**Authors:** Hadi Zare Marzouni, Reza Farid-Hosseini, Farahzad Jabari-Azad, Jalil Tavakkol-Afshari, Farahnaz Tehranian, Maryam Khoshkhui, Amin Reza Nikpoor, Mojgan Mohammadi

**Affiliations:** 1 *Allergy Research Center, Mashhad University of Medical Sciences, Mashhad, Iran.*; 2 *Immunology Research Center, Mashhad University of Medical Sciences, Mashhad, Iran.*; 3 *Research Centre of Iranian Blood Transfusion Organization, Khorasan Razavi, Mashhad, Iran.*

**Keywords:** Allergic Rhinitis, CD14, Polymorphism

## Abstract

**Introduction:**

Allergic Rhinitis (AR) is a common inflammatory disease of the nasal mucosa. The CD14 is a receptor for lipopolysaccharide and inhaled endotoxin which can stimulate the production of interleukins by antigen presenting cells. Accordingly, CD14 plays an important role in allergic and atopic diseases, which can be one of the etiological factors for allergic diseases. The present study investigated the association between the CD14 gene polymorphism C-159T and AR and aimed to detect the correlation between serum levels of CD14 and AR.

**Materials and Methods:**

This study was conducted on two groups of participants. The experimental group consisted of 125 patients with AR referring to Ghaem Hospital, Mashhad University of Medical Sciences in Mashhad, Iran, and the control group included 125 healthy subjects from Mashhad National Blood Center, Iran. Serum CD14 levels were measured by enzyme-linked immunosorbent assay. Polymerase chain reaction-restriction fragment length polymorphism was employed to detect C-159T gene polymorphism in the CD14 promoter region.

**Results:**

There was a significant association between CD14 C-159T gene polymorphism and AR (P<0.001). The results of the statistical analysis showed that the TT genotype could significantly increase the risk of AR (P<0.001). Additionally, a significant association was observed between C-159T gene polymorphism and the serum level of CD14 (P<0.001). Regardless of the genotypes, the serum CD14 levels were significantly higher in AR patients than in those of the participants in the controls (P=0.007).

**Conclusions:**

According to the obtained results of this study, CD14 in serum might be a potential marker for the diagnosis of AR, and in genetic levels it might be a predictive factor for the disease.

## Introduction

Allergic rhinitis (AR) is a type of inflammation of the mucous membrane in the nose, which affects 10-40% of people around the world (1-3). When sensitive people are exposed to allergens, an Immunoglobulin E-dependent (IgE-dependent) inflammation is formed. The symptoms of this disease usually include runny nose, continuous sneezing, irritation, itching, nasal congestion, lachrymation, headache, and problems with the senses of smell and taste, usually accompanied by conjunctivitis or inflammation of the cornea (1,3). The main cause of this disease is still unknown; however no one can ignore the influence of genetic and environmental factors (4-6). Many genetic studies have identified several chromosomal loci for this disease (6). There is a particular region in chromosome 5(5q31-32) which includes a set of cytokine genes, such as interleukin 4(IL-4), interleukin 13(IL-13), interleukin 9(IL-9), and granulocyte-macrophage-colony-timulating factor (GM-CSF). Each of these genes could play a significant role in the regulation of allergic responses (7). The CD14 gene which encodes a 55-kDa glycoprotein is located in 5q31-32. The CD14 is an important receptor for lipopolysaccharide (LPS) which can stimulate antigen presenting cells (APCs) to produce cytokines, such as TNF-alpha, IL-6, IL-8, and IL-12. Moreover, CD14 induces endocytosis pathway of toll-like receptor-4 (TLR-4) (8,9). Variations in the expression of the CD14 gene can influence IgE-dependent responses and inflammatory phenotype in patients with nasal polyposis (6). At the genetic level, the substitution of the nucleotide C with T is identified at the position of 159 in the promoter region of the CD14 gene. The protein level of CD14 is associated with the functional polymorphism, C-159T, in the CD14 gene (10). The CD14 also provides essential molecular signals for the receptor subunits of IL5, IL3, and GM-CSF in human eosinophils (7). Moreover, there is a relationship between CD14 C-159T gene polymorphism and some allergic diseases (10-14). Accordingly, this study aimed to investigate the serum levels of CD14 and AR patients, as well as analyzing the relationship between CD14 gene polymorphism C-159T and AR in a population selected from the city of Mashhad, located in northeastern Iran.

## Materials and Methods

This case-control study was conducted on 125 patients with AR referring to Ghaem Hospital, Mashhad University of Medical Sciences in 2014-2015. 

For the sample size, each of the case and control groups was supposed to have 105 individuals according to Yazdani et al. and the comparison of two population proportions formula (6). On the other hand, we increased the number to 125 participants in each group to ensure the suitability of the sample size. The error rates of α and β were 0.05 and 20%, respectively.

Patients signed a written consent form and participated in the study when their AR was confirmed by allergists. Inclusion criteria of the study were a diagnosis of AR and cutaneous sensitivity to local allergens. 

The exclusion criteria were a history of antihistamine usage, dermographism disease, and reluctance to participate due to personal reasons.

After patients were diagnosed with AR through clinical history-taking and skin prick tests, they completed 22-item Sinonasal Outcome Test (15). On the other hand, the control group consisted of 125 healthy individuals referring to National Blood Transfusion in Mashhad, Iran. The control subjects were selected from individuals who reported a negative history of certain diseases, such as malignancies, allergy, inflammatory, and autoimmune diseases. Additionally, routine blood screening tests were performed to detect human immunodeficiency virus, Hepatitis C virus, Hepatitis B virus, and Human T-cell leukemia virus type 1 in the blood of the control groups. Table 1 shows the clinical and demographic characteristics of patients with AR. The study began by collecting 10 ml of peripheral blood from each person and dividing it to two tubes. The first tube was used for serum separation to measure serum levels of CD14, which was kept at -80°C to be used for enzyme-linked immunosorbent assay (ELISA). The second tube contained Ethylenediami- netetraacetic acid (EDTA), which was used for the extraction of DNA and detection of CD14 gene polymorphism C-159T by employing the polymerase chain reaction-restriction fragment length polymorphism (PCR-RFLP) technique (6,16). 

*Skin prick test*

the skin prick test was performed using allergens, such as Salsola kali, grass mix, orchard grass, ash, Amaranthus retroflexus, lamb’s quarters, tree mix, Timothy, D. Farinae, D. Pteronyssinus, Bermuda grass, Kentucky grass, mugwort, willow, Alternaria, and Aspergillus mix allergens. Allergens were purchased from Greer Company (Mail: GREER®, PO Box 800 Lenoir, NC 28645). 

**Genomic DNA extraction and CD14 genotyping**

The PCR-RFLP technique was employed for the detection of CD14 gene polymorphism C-159T based on a previously published study (6). The products of RFLP were separated by electrophoresis in 1.5% agarose gel (Fig.2).

**Measurement of the serum levels of CD14**

Serum levels of CD14 were measured by ELISA according to the manufacturer’s instructions (sCD14 ELISA kit; Abcam, Cambridge, USA). 

**Statistical analysis**

Statistical analysis was performed using SPSS (version 16) through t-test and logistic regression tests. Hardy–Weinberg equilibrium was performed using the Chi-square test. The frequency of genotypes and allelotypes of C-159T gene polymorphism was calculated by direct counting. A P-value<0.05 was considered statistically significant. 

## Results

**Study population**

Patients with AR included 61 men and 64 women with a mean age of 34.67±10.04 years. On the other hand, the controls consisted of 61 men and 64 women with a mean age of 35.01±8.01 years. No significant difference was observed between patients with AR and the controls in terms of age (P=0.77) and gender (P=0.899). 

As can be seen in Table 1, except for itchy nose (P=0.039), there were no significant differences in other clinical manifestations between men and women (P>0.05). 

**Table 1:** Demographics and clinical characteristics of patients with AR.

**Variable**	**patients with AR n (%)**
**Gender**	
Male	61 (48.8%)
Female	64 (51.2%)
**Age**	
**Age (year)**	34.67±10.04
Range	18-55
**Family history of disease**	
Parents	32 (25.6%)
Siblings	35 (28%)
2nd and 3rd generation	22 (17.6%)
**History of comorbidities**	
Asthma	10 (8%)
Eczema	11 (8.8%)
Nose allergy	78 (62.4%)
Skin allergy	24 (19%)
Food allergy	44 (35.2%)
Drug allergy	4 (3.2%)
**Clinical symptoms**	
Rhinorrhea	114 (91.2%)
Nasal itching	70 (56%)
Nasal congestion	84 (67.2%)
Sneezing	105 (84%)
Shortness of breath	15 (12%)
Itchy eyes and throat	74 (59.2%)
Total	125

*Prick test*

 The results of the prick test demonstrated that the most frequent allergens were Salsola kali, lamb’s quarters, pigweed mix with the allergenicity of 90.4%, 82.4%, and 80.8%, respectively. Moreover, willow with 16.8% allergenicity was the least frequent allergen among patients in this study. 

The highest and lowest rates of skin inflammation were related to the Salsola kali (10.88±6.66 mm) and the willow (0.57±1.38 mm), respectively. Table 2 tabulates the allergenicity of all allergens.

**Table 2:** Serum CD14 levels, genotypes of CD14 polymorphism C-159T, and frequency of allergens in patients with AR

**Allergen**	**Male**		**Female**		**Max-Sensitive (mm)**	**Mean±SD** **(mm)**	**Genotype**			**P-value**		
	**n**	**%**	**N**	**%**			**CC** **(%)**	**CT (%)**	**TT (%)**	**P**_CC-CT_	**P**_CC-TT_	**P**_CT-TT_
**Salsola kali**	55	44	58	46.4	30	10.88±6.66	72.22	91.46	100	0.001	0.001	0.07
**Ash**	48	38.4	45	36	15	5.51±3.69	61.11	78.05	72	0.015	0.02	0.887
**Grass mix**	40	32	41	32.8	15	3.69±3.75	27.78	74.39	60	0.015	0.605	0.139
**Pigweed mix**	46	36.8	55	44	20	4.39±3.4	44.44	84.15	96	0.005	0.002	0.516
**Lambs quarter**	52	41.6	51	40.8	15	5.17±3.39	61.11	84.15	92	0.034	0.072	0.991
**Tree mix**	32	25.6	35	28	10	2.58±3.03	33.33	62.2	40	0.108	0.228	0.996
**Timothy**	23	18.4	16	12.8	15	1.67±3.11	27.78	26.83	48	0.889	0.545	0.641
**Orchard grass**	16	12.8	13	10.4	12	1.18±2.67	16.67	21.95	32	0.919	0.78	0.888
**Bermuda grass**	18	14.4	17	13.6	23	1.37±3	33.33	24.39	36	1.000	0.968	0.937
**Kentucky grass**	24	19.2	15	12	15	1.86±3.38	38.89	25.61	44	0.987	0.852	0.637
**Mugwort**	18	14.4	18	14.4	20	1.76±3.58	22.22	26.83	40	0.99	0.999	0.996
**Willow**	16	12.8	5	4	7	0.57±1.38	11.11	17.07	20	0.97	0.989	0.997
**D.Farinae**	23	18.4	25	20	6	1.25±1.76	33.33	41.46	32	0.98	0.704	0.183
**D.Pteronyssinus**	27	21.6	19	15.2	6	1.18±1.78	38.89	37.8	32	0.723	0.183	0.285
**Alternaria**	24	19.2	25	20	6	1.19±1.72	27.78	42.68	36	0.83	0.511	0.511
**Aspergillus Mix**	20	16	19	15.2	7	0.98±1.57	16.67	35.37	28	0.846	0.511	0.752

*Serum levels of CD14*

**Fig 1:**
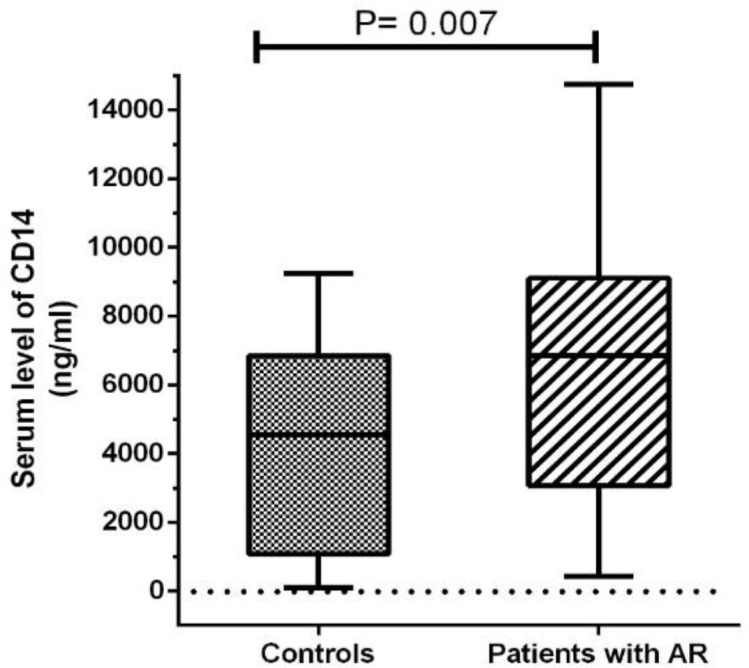
Median values and interquartile distances of serum levels of CD14 (ng/ml) in the case and control groups *CD14 Genotyping*

The mean serum levels of CD14 was 6237.2±3618.25 ng/ml in patients with AR and 4589.38±2907.22 ng/ml in controls. As shown in Figure 1, the serum levels of CD14 were significantly higher in patients with AR than in those in the control group (P=0.007). According to Table 2, the highest serum levels of CD14 in patients with AR were related to individuals who were sensitive to Alternaria, pigweed mix, and Salsola kali, respectively.

The genotype frequency for CD14 gene polymorphism C-159T in the control group was 42.4% (n=53) for the CC, 51.2% (n=64) for the CT, and 6.4% (n=8) for the TT genotypes. In patients with AR, it was 14.4% (n=18) for the CC, 65.6% (n=82) for the CT, and 20% (n=25) for the TT genotypes (Table 3). The genotype frequency for patients with AR was not in Hardy-Weinberg equilibrium. As can be seen in Table 3, there was a significant relationship between CD14 gene polymorphism C-159T and AR (P<0.001).

**Fig 2:**
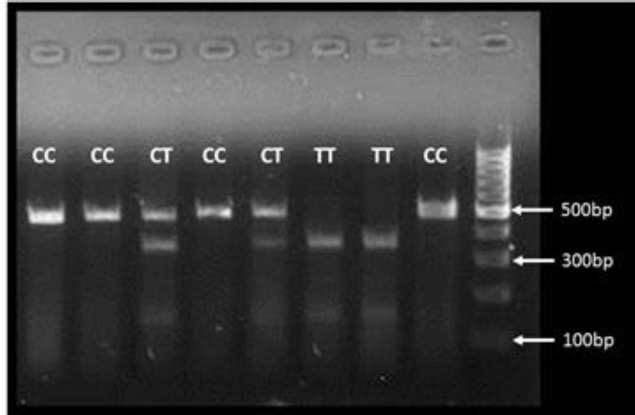
Image of 1.5% agarose gel for the detection of CD14 C-159T gene polymorphism using PCR-RFLP technique

**Table 3:** Frequency of CD14 genotypes C-159T and alleles in patients with AR and controls

**CD14 Genotype**	**patients with AR**		**Controls**		**OR**	**95% CI**	P-value	Allele	**patients with AR**		**Controls**	
	**N**	**%**	**N**	**%**					**N**	**%**	**N**	**%**
**CC**	18	14.4	53	42.4	1	Reference	<0.001	C	118	47.2	170	68
**CT**	82	65.6	64	51.2	3.773	2.016-7.06						
								T	132	52.8	80	32
**TT**	25	20	8	6.6	9.201	3.527-24.008						

**Fig 3:**
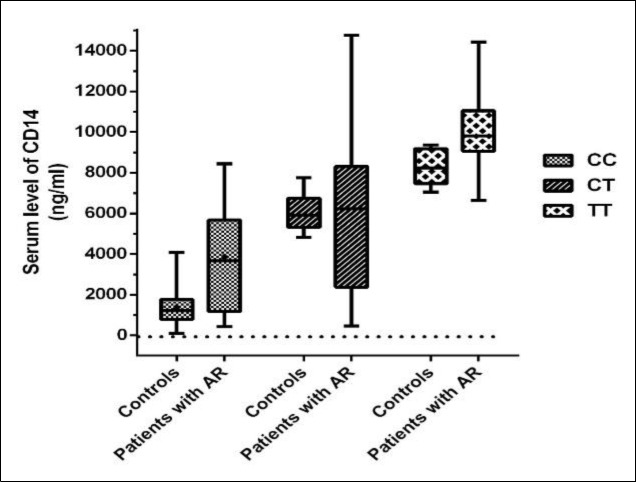
Serum levels of CD14 (ng/ml) in patients with AR and controls according to the CC, CT, and TT genotypes of CD14 C-159T polymorphism (in median values and interquartile distances)

Runny nose and sneeze were the most common clinical symptoms in 84% of people with the TT genotype. A significant difference was observed between patients with AR and clinical symptoms, including nasal congestion (P=0.034), sneezing (P=0.013), and wheezing (P=0.012). According to Table 2, the most frequent allergens in patients who had the TT genotype were Salsola kali (100%) and pigweed mix (96%). Moreover, there was a significant relationship between C-159T gene polymorphism and the serum level of CD14 (P<0.001). Serum CD14 levels in patients with AR and the controls were analyzed according to the CC, the CT, and the TT genotypes of CD14 polymorphism C-159T (Fig.3). As reported in Table 3, the risk of AR correlated significantly with the inheritance of TT genotype (P<0.001, OR: 9.201; 95% CI: 3.527-24.008).

## Discussion

The etiology of AR and other allergic diseases is related to factors that cause an excessive increase in IgE-dependent responses (1,3,4). Although the main cause of AR is still unknown, a number of studies have suggested that various molecular mechanisms are related to allergic responses (6). The major role of CD14 in the innate immune system and in immune responses has been reported in previous studies (6-9). CD14 can recognize LPS and other bacterial cell-wall components. 

Several studies investigated the relationship between a polymorphism named C-159T in the CD14 gene promoter and allergic diseases (6). The above-mentioned polymorphism showed a positive correlation with the serum levels of CD14, total IgE levels, and responses to the prick test (6,10,13,14). In an in vitro study, Hata et al. used transient transfection assays to study CD14 expression levels in monocytic cells. They concluded that C-159T polymorphism could increase the translation of CD14; therefore, it can be a functional gene polymorphism (17). Additionally, the results of luciferase reporter assays in the studies of LeVan et al. and Tahara et al. showed a functional effect of T allele on the serum levels of CD14 (18,19).

To the best knowledge of the researchers, this research is the first study which investigated the relationship between the functional polymorphism C-159T in the CD14 gene promoter region and serum CD14 levels in Iranian patients with AR. The results showed that C-159T polymorphism in the CD14 gene is highly associated with AR since AR was a significantly associated with TT and CT genotypes (P<0.001). The risk of AR in people with the TT and the CT genotypes is 9.2 and 3.8 times higher, respectively, compared to the individuals with the CC genotype. In other words, the risk of AR is higher in carriers of the T allele. 

Several previous studies showed a positive association between CD14 gene polymorphism C-159T and allergic diseases, as well as serum CD14 levels. Studies conducted by Gao et al. in England and Buckova et al. in the U.S. showed that the TT genotype was associated with the increase in the serum levels of CD14 and decrease in the serum levels of IgE in patients with allergic diseases such as asthma, allergic rhinitis (20,21), eczema, or chronic urticaria. Additionally, Augusto et al. reported a significant relationship between the above-mentioned gene polymorphism and the incidence of eczema (14).

In the studies performed by Baldini et al. and Demin Han et al. on patients with allergic diseases (10,22), the serum CD14 levels of individuals with the TT genotype were significantly higher than those of people with the CC and CT genotypes. Moreover, these studies reported a significant relationship between CD14 genotypes C-159T in patients who had positive prick tests compared to those who had negative prick tests. They concluded that CD14 gene polymorphism C-159T might play a significant role in the serum levels of CD14. Conversely, there is not always a significant relationship between C-159T gene polymorphism and atopic phenotypes. In some studies, no significant relationship was found between C-159T gene polymorphism and atopy in Hispanic populations or atopic asthma in Icelanders (23,24). Moreover, no significant relationship was observed between this polymorphism and asthma or atopy in German populations (25).

As Koppelman et al. reported CC genotype was associated with a decrease in positive prick test cases in Dutch people. In addition, they observed an association between carriers of the C allele in CD14 polymorphism C-159T and atopy in a Czechoslovakian population (26). The study of Leynaert et al. indicated that the risk of AR and atopy was lower in patients with the TT genotype than those with the CC genotype (13). However, the findings of studies on patients with allergic asthma were in contrast with those of AR patients. For instance, Yazdani et al. reported that the CC genotype was associated with the risk of asthma (6). Kowal et al. also showed that there was a relationship between CC genotype and the risk of disease in patients with house dust mite-allergic asthma (27). Additionally, Sharma et al. showed that the C allele was associated with the risk of atopic asthma (28).

These controversies might be due to differences in the pathophysiologies of asthma and AR. Additionally, the influence of gene-to-gene interactions and ethnic diversity might be other causative factors for this discrepancy. Differences in environmental conditions can also be considered as highly important factors in shaping such contraindications, as well. The interaction between CD14 gene polymorphism C-159T and environmental factors, such as smoking, pets and exposure to endotoxin, were reported in several studies (23,29-31). Moreover, Shweta et al., Eder et al. , Simpson, and Williams et al. reported a significant relationship between the level of endotoxin exposure and CD14 gene polymorphism C-159T in allergic sensitivity (23, 29-31). The studies carried out by Gern et al. (32) and Eder et al. (29) showed a positive relationship between living close to animals (domestic and farm ones) and and the above-mentioned polymorphism. They also showed a protective effect of the TT genotype on the individuals with low levels of exposure to domestic and farm animals. However, the same genotype was determined to be a risk factor in people with high levels of exposure to such animals. These results imply that various levels of exposure to microbial products in the environment might influence the interaction between genes and environmental factors and cause variations in the results of genetic studies regarding CD14 polymorphism C-159T.

The results of the current study also showed that the most frequent allergens in Mashhad were Salsola kali (90.4%) and lamb’s quarters (82.4%). Moreover, the maximum levels of inflammation in the skin prick test were observed for the above-mentioned allergens. Studies conducted by Barber et al. in Spain and Pereira et al. in Portugal reported similar findings(33,34). The study of Fereidouni et al. showed that during 2007-2009, Salsola kali with 72% of sensitivity was the first common allergen and pigweed mix was the second one in Mashhad (35). Additionally, Zare et al. reported that Salsola kali was the most frequent allergen in a population selected from the southern part of Iran (2). Akbari et al. found that Salsola kali, lamb’s quarters, and Egeria densa were the most frequent allergens in Isfahan, Iran (36). The results of the current study demonstrated that AR patients who were sensitive to Alternaria and pigweed mix had significantly higher serum CD14 levels compared to other allergens. Sahlander et al. investigated the relationship between serum CD14 levels and allergenic sensitivities, which resulted in a significant relationship between sensitivity to the studied allergens and serum levels of CD14 (37). However, Choon-Yee et al. reported that the serum levels of CD14 in patients sensitive to mites were lower than those of non-sensitive people (38).

This is the first time an association between sensitivity to Salsola kali, ash, and pigweed mix in patients with AR and CD14 polymorphism C-159T is being reported. Patients who had CT and TT genotypes showed significantly higher sensitivity to the above-mentioned allergens than those patients with the CC genotype. 

The results of the present study showed that CD14 in both protein levels and genetics might influence AR. 

Therefore, it is suggested that CD14 might be a serum immune biomarker for the diagnosis of AR. Additionally, it is suggested that CD14 gene polymorphism C-159T is a potential genetic predictive factor for AR. As a matter of fact, toll-like receptor (TLR) family, can recognize microbial products and be responsible for induction of both innate and adaptive immune responses. 

The TLR-4 is essential for the recognition of bacterial LPS and activation of signaling pathway. The signaling pathway is mediated by adaptor proteins, namely myeloid differentiation primary response 88, TIRAP, translocating chain-associated membrane protein, and TIR-domain-containing adapter-inducing interferon-β. These adaptor proteins are mediators between TLR-4 and transcription factors, such as NF-κB, AP-1 and Interferon Regulatory Factor-3, in order to produce proinflammatory cytokines (39,40). 

## Conclusion

We suggest to evaluate the expression of TLR-4 as well as serum levels of DC14 and total level of IgE in patients with AR for future studies. This might help us to find out the influence of CD14 on Th1 and Th2 balance, more precisely. Additionally, it might be helpful for future studies to divide patients with AR into atopic and non-atopic.

## References

[1] Scadding GK (2017). Allergic rhinitis: background, symptoms, diagnosis and treatment options. benefits.

[2] Zare Marzouni H, Akrami R, Shalilian M, Kalani N, Noori Ahmad Abadi M, Kooti W (2016). Investigating the Prevalence, Determining the Effects of Immunologic Sensitization and Clinical Symptoms Related to Allergens Existing in Khuzestan Province. Journal of Fasa University of Medical Sciences.

[3] Meltzer EO (2016). Allergic rhinitis: burden of illness, quality of life, comorbidities, and control. Immunology and allergy clinics of North America.

[4] Langdon C, Guilemany JM, Valls M, Alobid I, Bartra J, Dávila I (2016). Allergic rhinitis causes loss of smell in children: The OLFAPEDRIAL study. Pediatric Allergy and Immunology.

[5] Scadding GK, Scadding GW (2016). Diagnosing Allergic Rhinitis. Immunology and allergy clinics of North America.

[6] Yazdani N, Amoli M, Naraghi M, Mersaghian A, Firouzi F, Sayyahpour F (2012). 3 Association Between the Functional Polymorphism C-159T in the CD14 Promoter Gene and Nasal Polyposis: Potential Role in Asthma. Journal of Investigational Allergology and Clinical Immunology.

[7] Hamajima Y, Fujieda S, Sunaga H, Yamada T, Moribe K, Watanabe N (2007). Expression of Syk is associated with nasal polyp in patients with allergic rhinitis. Auris Nasus Larynx.

[8] Verhasselt V, Buelens C, Willems F, De Groote D, Haeffner-Cavaillon N, Goldman M (1997). Bacterial lipopolysaccharide stimulates the production of cytokines and the expression of costimulatory molecules by human peripheral blood dendritic cells: evidence for a soluble CD14-dependent pathway. The Journal of Immunology.

[9] Zanoni I, Ostuni R, Marek LR, Barresi S, Barbalat R, Barton GM (2011). CD14 controls the LPS-induced endocytosis of Toll-like receptor 4. Cell.

[10] Han D SW, Zhang L (2010). Association of the CD14 gene polymorphism C-159T with allergic rhinitis. Am J Rhinol Allergy.

[11] Keskin Ö, Birben E, Saçkesen C, Soyer ÖU, Alyamaç E, Karaaslan Ç (2006). The effect of CD14-c159T genotypes on the cytokine response to endotoxin by peripheral blood mononuclear cells from asthmatic children. Annals of Allergy, Asthma & Immunology.

[12] Kurne A, Sayat G, Aydin OF, Turgutoglu N, Terzi M, Sackesen C (2012). Lack of association of the CD14/ C− 159T polymorphism with susceptibility and progression parameters in Turkish multiple sclerosis patients. Journal of neuroimmunology.

[13] Leynaert B, Guilloud-Bataille M, Soussan D, Benessiano J, Guénégou A, Pin I (2006). Association between farm exposure and atopy, according to the CD14 C-159T polymorphism. Journal of allergy and clinical immunology.

[14] Litonjua AA, Belanger K, Celedón JC, Milton DK, Bracken MB, Kraft P (2005). Polymorphisms in the 5′ region of the CD14 gene are associated with eczema in young children. Journal of allergy and clinical immunology.

[15] Mortazavi H, Khalighi H, Goljanian A, Noormohammadi R, Mojahedi S, Sabour S (2015). Intra-oral low level laser therapy in chronic maxillary sinusitis: A new and effective recommended technique. Journal of clinical and experimental dentistry.

[16] Mohammadi M, Zahedi MJ, Nikpoor AR, Baneshi MR, Hayatbakhsh MM (2013). Interleukin-17 serum levels and TLR4 polymorphisms in ulcerative colitis. Iranian Journal of Immunology.

[17] Hata Y, Duh E, Zhang K, Robinson GS, Aiello LP (1998). Transcription factors Sp1 and Sp3 alter vascular endothelial growth factor receptor expression through a novel recognition sequence. Journal of Biological Chemistry.

[18] LeVan TD, Bloom JW, Bailey TJ, Karp CL, Halonen M, Martinez FD (2001). A common single nucleotide polymorphism in the CD14 promoter decreases the affinity of Sp protein binding and enhances transcriptional activity. The Journal of Immunology.

[19] Tahara T, Arisawa T, Shibata T, Hirata I, Nakano H (2007). Association of polymorphism of TLR4 and CD14 genes with gastroduodenal diseases in Japan. Inflammopharmacology.

[20] Gao PS, Mao XQ, Baldini M, Roberts M, Adra C, Shirakawa T (1999). Serum total IgE levels and CD14 on chromosome 5q31. Clinical genetics.

[21] Bučková D, Holla L, Schüller M, Znojil V, Vacha J (2003). Two CD14 promoter polymorphisms and atopic phenotypes in Czech patients with IgE‐mediated allergy. Allergy.

[22] Baldini M, Carla Lohman I, Halonen M, Erickson RP, Holt PG, Martinez FD (1999). A Polymorphism* in the 5′ flanking region of the CD14 gene is associated with circulating soluble CD14 levels and with total serum immunoglobulin E. American journal of respiratory cell and molecular biology.

[23] Choudhry S AP, Nazario S (2005). CD14 tobacco gene-environment interaction modifies asthma severity and immunoglobulin E levels in Latinos with asthma. Am J Respir Crit Care Med.

[24] Dennig J, Hagen G, Beato M, Suske G (1995). Members of the Sp transcription factor family control transcription from the uteroglobin promoter. Journal of Biological Chemistry.

[25] Sengler C, Haider A, Sommerfeld C, Lau S, Baldini M, Martinez F (2003). Evaluation of the CD14 C‐159 T polymorphism in the German Multicenter Allergy Study cohort. Clinical & Experimental Allergy.

[26] Koppelman GH, Reijmerink NE, Colin Stine O, Howard TD, Whittaker PA, Meyers DA (2001). Association of a promoter polymorphism of the CD14 gene and atopy. American journal of respiratory and critical care medicine.

[27] Kowal K, Bodzenta-Lukaszyk A, Pampuch A, Szmitkowski M, Zukowski S, Donati MB (2008). Analysis of -675 4 g/5 G SERPINE1 and C-159T CD14 polymorphisms in house dust mite-allergic asthma patients. Journal of investigational allergology & clinical immunology.

[28] Sharma M, Batra J, Mabalirajan U, Goswami S, Ganguly D, Lahkar B (2004). Suggestive evidence of association of C-159T functional polymorphism of the CD14 gene with atopic asthma in northern and northwestern Indian populations. Immunogenetics.

[29] Eder W, Klimecki W, Yu L, Von Mutius E, Riedler J, Braun-Fahrländer C (2005). Opposite effects of CD14/-260 on serum IgE levels in children raised in different environments. Journal of Allergy and Clinical Immunology.

[30] Simpson A, John SL, Jury F, Niven R, Woodcock A, Ollier WE (2006). Endotoxin exposure, CD14, and allergic disease: an interaction between genes and the environment. American journal of respiratory and critical care medicine.

[31] Williams LK, McPhee RA, Ownby DR, Peterson EL, James M, Zoratti EM (2006). Gene-environment interactions with CD14 C-260T and their relationship to total serum IgE levels in adults. Journal of Allergy and Clinical Immunology.

[32] Gern JE, Reardon CL, Hoffjan S, Nicolae D, Li Z, Roberg KA (2004). Effects of dog ownership and genotype on immune development and atopy in infancy. Journal of Allergy and Clinical Immunology.

[33] Barber D, De La Torre F, Feo F, Florido F, Guardia P, Moreno C (2008). Understanding patient sensitization profiles in complex pollen areas: a molecular epidemiological study. Allergy.

[34] Pereira C, Valero A, Loureiro C, Davila I, Martinez-Cocera C, Murio C (2006). Iberian study of aeroallergens sensitisation in allergic rhinitis. European annals of allergy and clinical immunology.

[35] Fereidouni M, Hossini RF, Azad FJ, Assarezadegan MA, Varasteh A (2009). Skin prick test reactivity to common aeroallergens among allergic rhinitis patients in Iran. Allergologia et immunopathologia.

[36] Akbari H RA (2000). Common allergens for allergic patients in Isfahan: A clinically-based study. Journal of Research In Medical Sciences.

[37] Sahlander K, Larsson K, Palmberg L (2010). Increased serum levels of soluble ST2 in birch pollen atopics and individuals working in laboratory animal facilities. Journal of occupational and environmental medicine.

[38] Tan C-Y, Chen Y-L, Wu LS-H, Liu C-F, Chang W-T, Wang J-Y (2006). Association of CD14 promoter polymorphisms and soluble CD14 levels in mite allergen sensitization of children in Taiwan. Journal of human genetics.

[39] Kagan JC, Su T, Horng T, Chow A, Akira S, Medzhitov R (2008). TRAM couples endocytosis of Toll-like receptor 4 to the induction of interferon-β. Nature immunology.

[40] Akira S, Takeda K (2004). Toll-like receptor signalling. Nature reviews immunology.

